# Evaluation of the Performance of the Cobas CT/NG Test for Use on the Cobas 6800/8800 Systems for Detection of *Chlamydia trachomatis* and *Neisseria gonorrhoeae* in Male and Female Urogenital Samples

**DOI:** 10.1128/JCM.01996-18

**Published:** 2019-03-28

**Authors:** Barbara Van Der Pol, Kenneth Fife, Stephanie N. Taylor, Melinda B. Nye, Steven E. Chavoustie, David L. Eisenberg, LaShonda Crane, Gregory Hirsch, Rodney Arcenas, Elizabeth M. Marlowe

**Affiliations:** a University of Alabama at Birmingham, Birmingham, Alabama, USA; b Indiana University School of Medicine, Indianapolis, Indiana, USA; c Louisiana State University, New Orleans, Louisiana, USA; d Laboratory Corporation of America Holdings, Burlington, North Carolina, USA; e Healthcare Clinical Data, Inc., North Miami, Florida, USA; f Planned Parenthood of St. Louis Region and Southwest Missouri, St. Louis, Missouri, USA; g Planned Parenthood Gulf Coast, Inc., Houston, Texas, USA; h Planned Parenthood of Northern, Central and Southern New Jersey, Inc., Morristown, New Jersey, USA; i Roche Molecular Systems, Inc., Pleasanton, California, USA; Marquette University

**Keywords:** *Chlamydia trachomatis*, *Neisseria gonorrhoeae*, PCR, Cobas CT/NG assay, genital infection, molecular diagnostics, nucleic acid amplification test, sexually transmitted infection

## Abstract

The clinical performance of the Cobas CT/NG assay on the Cobas 6800/8800 systems (Cobas) for the detection of Chlamydia trachomatis and Neisseria gonorrhoeae was established in a multisite, prospective collection study using male and female urogenital specimens; supportive data from archived specimens were also included. The results obtained with the Cobas assay were compared with the patient infected status derived from a combination of U.S. Food and Drug Administration-approved nucleic acid amplification tests to determine the sensitivity and specificity of detection from each sample type. The sensitivity of Cobas for the detection of C. trachomatis in female specimens was 95.6% (95% confidence interval [CI], 92.4% to 97.4%) for urine; 98.6% (95% CI, 95.2% to 99.6%) and 99.2% (95% CI, 95.4% to 99.9%) for clinician- and self-collected vaginal swab specimens, respectively; 93.3% (95% CI, 89.6% to 95.7%) for endocervical swabs; and 92.5% (95% CI, 88.7% to 95.1%) for cervical swab samples in PreservCyt. The specificity for the detection of C. trachomatis was ≥98.8% for all female sample types. Sensitivity and specificity estimates of Cobas for the detection of C. trachomatis in male urine samples were 100% (96.8% to 100.0%) and 99.7% (95% CI, 99.2% to 99.9%), respectively. The sensitivity of Cobas for the detection of N. gonorrhoeae in female specimens was 94.8% (95% CI, 89.6% to 97.4%) for urine; 100.0% (95% CI, 87.9% to 100.0%) and 100.0% (95% CI, 87.9% to 100.0%) for clinician- and self-collected vaginal swab specimens, respectively; 97.0% (95% CI, 91.5% to 99.0%) for endocervical swabs; and 96.6% (95% CI, 90.6% to 98.8%) for cervical samples in PreservCyt; the specificity for all female sample types was >99.0%. The sensitivity and specificity of Cobas for detecting N. gonorrhoeae in male urine were 100.0% (95% CI, 95.8% to 100.0%) and 99.5% (95% CI, 98.8% to 99.8%), respectively. Fully automated assays help fill the clinical need for a sensitive, high-throughput screening tool to aid public health efforts to control C. trachomatis and N. gonorrhoeae infections.

## Abstract (precis)

The clinical performance of the Cobas CT/NG assay on the Cobas 6800/8800 systems (Cobas) for the detection of Chlamydia trachomatis and Neisseria gonorrhoeae was established in a multisite, prospective collection study using male and female urogenital specimens; supportive data from archived specimens were also included. The results obtained with the Cobas assay were compared with the patient infected status derived from a combination of U.S.

## INTRODUCTION

Chlamydia trachomatis and Neisseria gonorrhoeae are the leading bacterial causes of sexually transmitted infections (STIs) worldwide, with an estimated 131 million and 78 million new cases, respectively, each year (1). In the United States, approximately 1.7 million cases of C. trachomatis infection and 555,608 cases of N. gonorrhoeae infection were reported in 2017, representing increases of 4.7% and 18.5%, respectively, since 2015 (2). Both C. trachomatis and N. gonorrhoeae are major causes of pelvic inflammatory disease, ectopic pregnancy, and tubal factor infertility, and they facilitate the acquisition and transmission of human immunodeficiency virus (3–6). The importance of routine screening for C. trachomatis and N. gonorrhoeae to reduce the risk of these complications is well established (4, 7–10), and countries worldwide have implemented routine screening guidelines for populations with the highest risk of infection (11–15). Screening programs have been improved by expansion of acceptable sample types, such as self-collected vaginal swab samples, which have been shown to be equivalent in performance to clinician-collected samples and to be well accepted by patients (16, 17). The increasing use of preexposure prophylaxis (PrEP) for human immunodeficiency virus in high-risk individuals adds to the demand for routine STI screening programs (18–21); the Centers for Disease Control and Prevention (CDC) recommendation of quarterly STI screening for individuals on PrEP increases the demands of high-volume screening programs. These guidelines specifically include testing for C. trachomatis and N. gonorrhoeae for those sexually active adults that are prescribed PrEP. Furthermore, with shifting health care provision options and reduced funding for STI-specific health care clinics, offering STI screening in primary health care settings, such as general medicine, family planning, and obstetrics/gynecology clinics, is becoming more common. Diagnostic solutions that support the increasing need for STI screening across multiple populations and health care settings are clearly relevant to today’s STI control efforts.

The CDC STI laboratory diagnostics recommendations (22) state that infection with C. trachomatis or N. gonorrhoeae should be detected using highly sensitive and specific nucleic acid amplification tests (NAATs). High-throughput NAATs can support national and community-based screening programs, leading to the earlier detection and treatment of infections, reduced transmission and prevalence, and reduced complications and associated health care costs (10, 22–25).

While automated STI screening solutions exist, a substantial need remains for fully automated, high-throughput molecular assays to address the increasing demands for STI screening services (26, 27). In this study, we evaluated the Cobas CT/NG test for use on the Cobas 6800/8800 systems (Cobas), which may address the needs of high-throughput laboratories (28, 29). The objective of this study was to evaluate the clinical performance of Cobas in urogenital samples from symptomatic and asymptomatic men and women.

## MATERIALS AND METHODS

### Patient population.

Samples were prospectively collected from participants attending 8 geographically diverse family planning, obstetrics/gynecology, and STI clinics in the United States (Houston, TX; Miami, FL; Indianapolis, IN; New Orleans, LA; Seattle, WA; Morristown, NJ; St. Louis, MO; and Birmingham, AL). Eligible participants were aged ≥14 years, had been sexually active within the past 6 months, and were willing and able to provide written informed consent. Individuals were excluded if any of the following conditions were met: (i) they had previously been enrolled in the study; (ii) they had received antimicrobial therapy during the previous 21 days; (iii) they had used Replens (Lil’Drug Store Products, Inc., Cedar Rapids, IA), RepHresh odor-eliminating vaginal gel or RepHresh clean balance (Church & Dwight, Co, Inc., Princeton, NJ), or products containing metronidazole within the previous 3 days; (iv) they had a history of a full hysterectomy; or (v) they had a contraindication to Papanicolaou testing or cervical sampling. Participants were classified as symptomatic if they self-reported at least one of the following symptoms: dysuria; coital pain, postcoital bleeding; abnormal vaginal discharge or bleeding; pelvic, uterine, or ovarian pain; urethral discharge; testicular pain; and scrotal pain or swelling. All other participants were classified as asymptomatic. This study was conducted in compliance with the International Conference on Harmonisation good clinical practice guidelines and the regulations of the U.S. Food and Drug Administration (FDA). Institutional review board approval was obtained from each participating study site prior to the start of the study.

Due to the low prevalence of N. gonorrhoeae among women, a subset of archived samples collected in support of the U.S. clinical trial for the Cobas CT/NG assay on the Cobas 4800 system (Cobas 4800) (30–32) was retested on Cobas (6800/8800 systems), and the results were included in the analysis.

### Specimen collection.

The following specimens were collected (in order) from women: 1 first-catch urine (FCU) specimen; 1 self-collected (SC) vaginal swab specimen placed in Cobas PCR medium (from approximately 50% of participating women); up to 3 clinician-collected (CC) vaginal swab specimens; 1 endocervical swab specimen; and 1 cervical swab sample in PreservCyt solution, with collection of these samples alternating between the use of a spatula/cytobrush and a broom. The comparator assay vaginal swab collection order was randomized and was performed by clinicians. For women not randomized to self-obtain the Cobas vaginal swab specimen, clinicians collected this last among all of the vaginal swab samples collected. Archived sample types for the N. gonorrhoeae analyses included FCU, samples in PreservCyt, and endocervical swabs. One FCU sample was collected from each participating man.

### Specimen testing.

The Cobas CT/NG test is a qualitative nucleic acid amplification test performed on the Cobas 6800/8800 system; the system’s features have been previously described in detail (28, 29). Prospectively collected samples from each participant were tested with Cobas at 1 of 3 test sites. The archived samples were tested internally at Roche Molecular Systems, Pleasanton, CA. The FDA-approved NAATs used to determine patient infected status (PIS) included the BD ProbeTec CT Q^x^ and GC Q^x^ amplified DNA assays (CTQ/GCQ; BD, Sparks, MD), the Hologic Aptima Combo 2 CT/NG assay (AC2; Hologic Inc., San Diego, CA), and the Abbott m2000 RealTi*m*e CT/NG assay (m2000; Abbott Molecular, Des Plaines, IL). m2000 was used only for male urine testing to provide a third result. For all systems, samples were tested and data were interpreted according to the respective manufacturers’ instructions for use.

### Patient infected status for C. trachomatis and N. gonorrhoeae.

Four comparator test results were available for women: urine and vaginal swab specimen results from both CTQ/GCQ and AC2. Women were categorized as being infected with C. trachomatis or N. gonorrhoeae (PIS) if both NAATs (CTQ/GCQ and AC2) generated positive results in at least one sample type (urine and/or vaginal swab specimens). Endocervical swab and PreservCyt specimens were tested only with Cobas and were therefore not included in the calculation of the PIS. The endocervical and PreservCyt test results were compared with the PIS of women. Men were classified as infected if 2 of 3 NAATs (CTQ/GCQ, AC2, and m2000) gave positive results using FCU. Each NAAT was also evaluated using a rotating PIS analysis, constructed from the results obtained from the other NAATs used in the study, including Cobas results, based on the above-mentioned criteria for defining PIS. The rotating PIS analyses incorporated the results from the prospectively collected specimens only.

### Data analysis and interpretation of results.

All data analyses were performed using SAS/STAT software. The sensitivity, specificity, positive predictive value (PPV), and negative predictive value (NPV) of Cobas were calculated overall and by symptom status for detection of C. trachomatis and N. gonorrhoeae compared with the PIS for each sex (see Table S1 in the supplemental material). Specimens with invalid test results and/or for which further retests could not be performed due to a lack of sample volume were excluded from the data analyses.

The sensitivity, specificity, PPV, and NPV of Cobas were estimated separately for each sample type and symptom status. Two-sided 95% confidence intervals (CIs) were calculated using the Wilson method for proportions. Positive percent agreement (PPA), negative percent agreement (NPA), and overall percent agreement (OPA) were assessed by pairwise comparison between Cobas and the comparator tests with matched clinical specimen types (i.e., the same sample type from women). For the comparison of performance between assays, the Fisher exact test was performed, and *P* values are presented.

## RESULTS

### Patient population.

**FIG 1:**
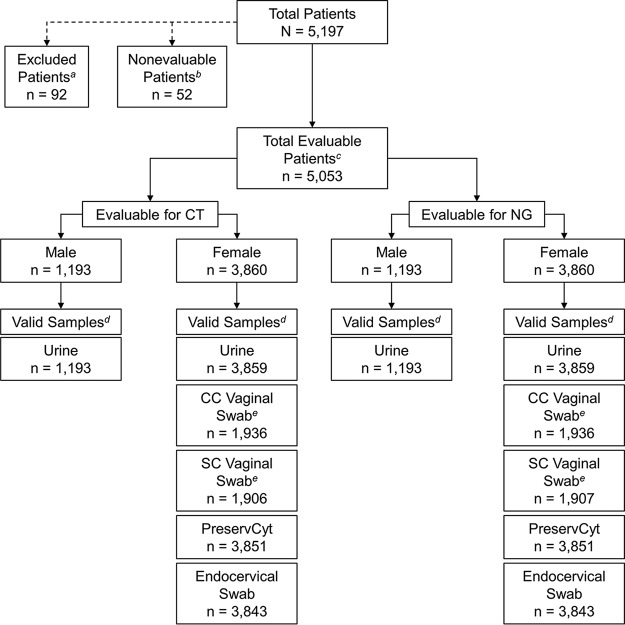
Patient disposition and specimen evaluability in the prospective population. *^a^*, reasons for exclusion included clinician decision (*n* = 3), the patient did not meet the eligibility criteria (*n* = 21), the patient was unwilling to provide informed consent (*n* = 37), pregnancy (*n* = 1), protocol deviation (*n* = 14), screen failure (*n* = 1), and withdrawal by the patient (*n* = 15); *^b^*, nonevaluable subjects were those without an assigned C. trachomatis/N. gonorrhoeae patient infected status (PIS; *n* = 51) or invalid/failed Cobas test results (*n* = 1). *^c^*, patients were considered evaluable if they had both a designated C. trachomatis/N. gonorrhoeae PIS, based on the prespecified PIS algorithm, and valid Cobas results for at least one sample type; *^d^*, valid samples were those with valid Cobas results and were included in the analysis; samples with missing, invalid, or failed Cobas results were excluded from the analysis; *^e^*, prospectively enrolled women were randomly assigned to the clinician-collected (CC) or self-collected (SC) vaginal swab specimen group. CT, C. trachomatis; NG, N. gonorrhoeae.

A total of 5,197 participants were enrolled in the prospective population; of these, 92 were excluded because they did not meet the inclusion criteria and 52 were excluded due to invalid test results and/or the inability to retest specimens (Fig. 1). A total of 5,053 participants were evaluable and included in the analyses. Archived samples from an additional 295 women were included in the N. gonorrhoeae analyses. Patient demographics and baseline characteristics are shown in Table 1. Overall, there were 16,587 C. trachomatis test results (prospective population) and 16,959 N. gonorrhoeae (prospective and archived population) test results that were included in the analyses.

**TABLE 1:** Patient demographics and baseline characteristics

Characteristic	Values for:	
	Prospective population^*a*^ (*n* = 5,053)	Archived samples^*b*^ (*n* = 295)
Age (yr)		
Mean (SD)	30.4 (9.8)	27.5 (9.2)
Median (range)	28 (14–79)	25 (18–70)
No. (%) of subjects by sex		
Female	3,860 (76.4)	295 (100)
Male	1,193 (23.6)	0
No. (%) of subjects by race		
Black/African American	2,843 (49.1)	159 (53.9)
White	1,946 (38.5)	116 (39.3)
Asian	91 (1.8)	6 (2.0)
American Indian/Alaska Native	26 (0.5)	0
Native Hawaiian/Pacific Islander	8 (0.2)	1 (0.3)
Multiple/other	323 (6.4)	13 (4.4)
Not reported	176 (3.5)	0
No. (%) of subjects by ethnicity		
Not Hispanic/Latino	3,541 (70.1)	226 (76.6)
Hispanic/Latino	1,379 (27.3)	58 (19.7)
Unknown	52 (1.0)	2 (0.7)
Not reported	81 (1.6)	9 (3.1)
No. (%) of subjects by self-reported symptom status		
Asymptomatic	3,305 (65.4)	145 (49.2)
Symptomatic	1,747 (34.6)	150 (50.8)
No. (%) of subjects by pregnancy status (women only)		
Not pregnant	3,811 (98.7)	281 (95.3)
Pregnant	43 (1.1)	9 (3.1)
Unknown	6 (0.2)	5 (1.7)
No. (%) of subjects by clinic type		
Family planning	3,130 (61.9)	134 (45.4)
STI^*c*^	973 (19.3)	120 (40.7)
Obstetrics/gynecology	797 (15.8)	41 (13.9)
Family planning/STI	153 (3.0)	0

a Participants enrolled in the prospective study population with designated infection status (infected or not infected) and final valid Cobas CT/NG test results.

b Archived samples from study COB-CTNG-282 from female participants with a designated N. gonorrhoeae PIS (infected or not infected), final valid Cobas CT/NG test results, and an adequate sample volume for testing.

c STI, sexually transmitted infection.

### Chlamydia trachomatis.

**FIG 2:**
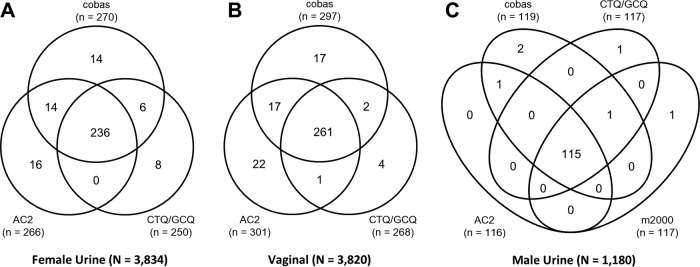
Venn diagrams comparing C. trachomatis-positive results across NAATs with urine samples from women (A), vaginal swab samples (B), and urine samples from men (C).

Of the 3,860 evaluable, prospectively enrolled women, 3,834 had valid urine sample results and 3,820 had valid vaginal swab sample results with Cobas, AC2, and CTQ/GCQ; 1,180 of 1,193 evaluable, prospectively enrolled men had valid urine sample results with Cobas, AC2, CTQ/GCQ, and m2000. In total, 271 (7.0%) of 3,860 women in the prospective population were defined as C. trachomatis infected based on the PIS. Sensitivity and specificity by sample type are shown in Table 2. Overall the sensitivity and specificity of C. trachomatis detection for all female sample types were ≥92.5 and ≥98.8%, respectively. A total of 118 (9.9%) of 1,193 men were defined as being infected with C. trachomatis, with sensitivity and specificity estimates for Cobas of 100% (95% CI, 96.8% to 100.0%) and 99.7% (95% CI, 99.2% to 99.9%), respectively. Venn diagrams comparing the C. trachomatis-positive results across all NAATs, regardless of PIS, in female urine, female vaginal swab, and male urine samples are shown in Fig. 2. The OPA between Cobas and each predicate NAAT for the detection of C. trachomatis in all sample types was >98%, with a PPA of >92% and an NPA of >99%.

**TABLE 2:** Clinical performance of Cobas versus PIS by symptomatic status for C. trachomatis^*a*^

Sex, sample type	Symptom status	Total	% sensitivity (no. of samples positive/total no. tested)	95% CI for sensitivity	% specificity (no. of samples positive/total no. tested)	95% CI	Prevalence (%)	PPV (%)	NPV (%)
Women									
Urine	Symptomatic	1,441	96.0 (119/124)	90.9–98.3	99.8 (1,315/1,317)	99.4–100.0	8.6	98.3	99.6
	Asymptomatic	2,418	95.2 (140/147)	90.5–97.7	99.6 (2,262/2,271)	99.2–99.8	6.1	94.0	99.7
	Overall	3,859	95.6 (259/271)	92.4–97.4	99.7 (3,577/3,588)	99.5–99.8	7.0	95.9	99.7
CC vaginal swab	Symptomatic	711	100.0 (63/63)	94.3–100.0	99.2 (643/648)	98.2–99.7	8.9	92.6	100.0
	Asymptomatic	1,225	97.6 (83/85)	91.8–99.4	99.0 (1,129/1,140)	98.3–99.5	6.9	88.3	99.8
	Overall	1,936	98.6 (146/148)	95.2–99.6	99.1 (1,772/1,788)	98.6–99.4	7.6	90.1	99.9
SC vaginal swab	Symptomatic	720	100.0 (59/59)	93.9–100.0	98.8 (653/661)	97.6–99.4	8.2	88.1	100.0
	Asymptomatic	1,186	98.4 (60/61)	91.3–99.7	99.2 (1,116/1,125)	98.5–99.6	5.1	87.0	99.9
	Overall	1,906	99.2 (119/120)	95.4–99.9	99.0 (1,769/1,786)	98.5–99.4	6.3	87.5	99.9
PreservCyt	Symptomatic	1,438	95.1 (116/122)	89.7–97.7	99.5 (1,309/1,316)	98.9–99.7	8.5	94.3	99.5
	Asymptomatic	2,413	90.3 (131/145)	84.4–94.2	99.7 (2,261/2,268)	99.4–99.9	6.0	94.9	99.4
	Overall	3,851	92.5 (247/267)	88.7–95.1	99.6 (3,570/3,584)	99.3–99.8	6.9	94.6	99.4
Endocervical swab	Symptomatic	1,433	95.9 (116/121)	90.7–98.2	99.1 (1,300/1,312)	98.4–99.5	8.4	90.6	99.6
	Asymptomatic	2,410	91.1 (133/146)	85.4–94.7	99.5 (2,253/2,264)	99.1–99.7	6.1	92.4	99.4
	Overall	3,843	93.3 (249/267)	89.6–95.7	99.4 (3,553/3,576)	99.0–99.6	6.9	91.5	99.5
Men, urine	Symptomatic	305	100.0 (63/63)	94.3–100.0	99.6 (241/242)	97.7–99.9	20.7	98.4	100.0
	Asymptomatic	887	100.0 (55/55)	93.5–100.0	99.8 (830/832)	99.1–99.9	6.2	96.5	100.0
	Overall	1,192	100.0 (118/118)	96.8–100.0	99.7 (1,071/1,074)	99.2–99.9	9.9	97.5	100.0
Overall		16,587	95.5 (1,138/1,191)	94.2–96.6	99.5 (15,312/15,396)	99.3–99.6	7.2	93.1	99.7

a CC, clinician collected; Cobas, Cobas CT/NG test for use on the Cobas 6800/8800 systems; NPV, negative predictive value; PIS, patient infected status; PPV, positive predictive value; PreservCyt, cervical sample collected in PreservCyt cytology medium; SC, self-collected.

Overall, there were 1,936 CC and 1,906 SC prospective vaginal swab specimens. The Cobas sensitivity performances for the detection of C. trachomatis from CC and SC vaginal swabs were very similar at 98.6% (95% CI, 95.2% to 99.6%) and 99.2% (95% CI, 95.4% to 99.9%), respectively. The specificity performances were also very similar at 99.1% (95% CI, 98.6% to 99.4%) and 99.0% (95% CI, 98.5% to 99.4%), respectively (Table 2).

The sensitivity and specificity for C. trachomatis detection with Cobas, AC2, CTQ/GCQ, and m2000 (male urine only) were estimated using a rotating PIS analyses that incorporated the results obtained with prospectively collected female urine, female vaginal swab, and male urine specimens. The sensitivity of the Cobas assay was significantly higher than that of CTQ/GCQ for female urine (*P* < 0.001) and vaginal swabs (*P = *0.006) (Table 3). The specificities of all assays for the detection of C. trachomatis in both female urine and vaginal swab samples were ≥99.0% across the rotating PIS assay combinations (Table 3). The sensitivities and specificities for the detection of C. trachomatis in male urine samples were comparable with Cobas, AC2, CTQ/GCQ, and m2000 (≥98.3% and ≥99.7%, respectively; Table 4).

**TABLE 3:** Rotating PIS analysis of sensitivity and specificity of NAATs for detecting Chlamydia trachomatis in urine and clinician-collected vaginal swab samples from women^*a*^

Comparison, sample type	No. of samples tested	No. of C. trachomatis PIS-positive samples tested/total no.	% sensitivity (95% CI)	*P* value for comparison with Cobas sensitivity	No. of C. trachomatis PIS-negative samples tested/total no.	% specificity (95% CI)	*P* value for comparison with Cobas specificity
AC2 vs PIS (Cobas and CTQ/GCQ)							
Urine from women	3,839	254/272	93.4 (89.8–95.8)	0.348	3,555/3,567	99.7 (99.4–99.8)	0.838
Vaginal swab (clinician)	3,836	266/270	98.5 (96.3–99.4)	1.000	3,531/3,566	99.0 (98.6–99.3)	0.882
CTQ/GCQ vs PIS (Cobas and AC2)							
Urine from women	3,852	243/285	85.3 (80.7–88.9)	<0.001	3,560/3,567	99.8 (99.6–99.9)	0.480
Vaginal swab (CC)	3,825	262/283	92.6 (88.9–95.1)	0.006	3,536/3,542	99.8 (99.6–99.9)	<0.001

a AC2, Hologic Aptima Combo 2 CT/NG assay; CC, clinician collected; Cobas, Cobas CT/NG test for use on the Cobas 6800/8800 systems; CTQ/GCQ, BD ProbeTec CT Q^x^ and GC Q^x^ amplified DNA assays; NAAT, nucleic acid amplification test; PIS, patient infected status.

**TABLE 4:** Rotating PIS analysis of sensitivity and specificity of NAATs for detecting Chlamydia trachomatis in urine samples from men^*a*^

Comparison	No. of samples tested	No. of C. trachomatis PIS-positive samples tested/total no.	% sensitivity (95% CI)	No. of C. trachomatis PIS-negative samples tested/total no.	% specificity (95% CI)
AC2 vs PIS (Cobas, CTQ/GCQ, and m2000)	1,187	117/119	98.3 (94.1–99.5)	1,067/1,068	99.9 (99.5–100.0)
CTQ/GCQ vs PIS (Cobas, AC2, and m2000)	1,187	116/117	99.1 (95.3–99.8)	1,069/1,070	99.9 (99.5–100.0)
m2000 vs PIS (Cobas, AC2, and CTQ/GCQ)	1,189	118/119	99.2 (95.4–99.9)	1,069/1,070	99.9 (99.5–100.0)

a AC2, Hologic Aptima Combo 2 CT/NG assay; Cobas, Cobas CT/NG test for use on the Cobas 6800/8800 systems; CTQ/GCQ, BD ProbeTec CT Q^x^ and GC Q^x^ amplified DNA assays; m2000, Abbot m2000 RealTi*m*e CT/NG assay; NAAT, nucleic acid amplification test; PIS, patient infected status.

### Neisseria gonorrhoeae.

**FIG 3:**
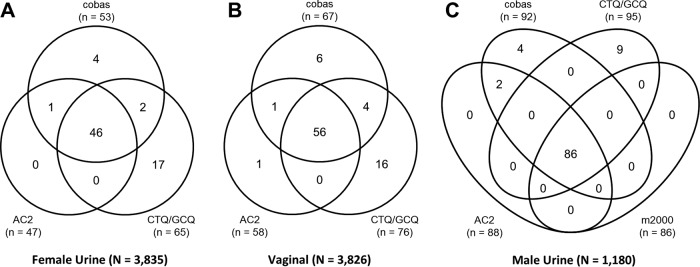
Venn diagrams comparing N. gonorrhoeae-positive results across NAATs with urine samples from women (A), vaginal swab samples (B), and urine samples from men (C) (prospective population).

Of the 3,860 evaluable, prospectively enrolled women, 3,835 had valid urine sample results and 3,826 had valid vaginal swab sample results on Cobas, AC2, and CTQ/GTQ; 1,180 of 1,193 evaluable, prospectively enrolled men had valid urine sample results on Cobas, AC2, CTQ/GTQ, and m2000. Of the 3,860 prospectively enrolled women, 57 (1.5%) were considered N. gonorrhoeae infected based on the PIS; of the 295 women who contributed archived specimens, 82 were N. gonorrhoeae infected. Sensitivity and specificity by sample type are shown in Table 5. Overall, among the prospective and archived female specimens, the sensitivity and specificity of Cobas for detecting N. gonorrhoeae were ≥94.8% and ≥99.7%, respectively. A total of 87 (7.3%) of 1,193 men were considered N. gonorrhoeae infected based on the PIS, with sensitivity and specificity estimates of Cobas for the detection of N. gonorrhoeae in male urine of 100.0% (95% CI, 95.8% to 100.0%) and 99.5% (95% CI, 98.8% to 99.8%), respectively. Venn diagrams comparing the N. gonorrhoeae-positive results across all NAATs, regardless of PIS, in female urine, female vaginal swab, and male urine samples are shown in Fig. 3. The OPA between Cobas and each predicate NAAT for N. gonorrhoeae in all sample types was >98%, with a PPA of >73% and an NPA of >99%.

**TABLE 5:** Clinical performance of Cobas versus PIS by symptomatic status for N. gonorrhoeae^*a*^

Sex, sample type	Symptom status	Total	% sensitivity (no. of samples positive/total no. tested)	95% CI for sensitivity	% specificity (no. of samples positive/total no. tested)	95% CI for specificity	Prevalence (%)	PPV (%)	NPV (%)
Women									
Urine	Symptomatic	1,535	96.7 (59/61)	88.8–99.1	99.8 (1,471/1,474)	99.4–99.9	4.0	95.2	99.9
	Asymptomatic	2,519	93.2 (68/73)	84.9–97.0	100.0 (2,446/2,446)	99.8–100.0	2.9	100.0	99.8
	Overall	4,054	94.8 (127/134)	89.6–97.4	99.9 (3,917/3,920)	99.8–100.0	3.3	97.7	99.8
CC vaginal swab	Symptomatic	711	100.0 (11/11)	74.1–100.0	99.7 (698/700)	99.0–99.9	1.5	84.6	100.0
	Asymptomatic	1,225	100.0 (17/17)	81.6–100.0	99.8 (1,205/1,208)	99.3–99.9	1.4	85.0	100.0
	Overall	1,936	100 (28/28)	87.9–100.0	99.7 (1,903/1,908)	99.4–99.9	1.4	84.8	100.0
SC vaginal swab	Symptomatic	720	100.0 (14/14)	78.5–100.0	99.7 (704/706)	99.0–99.9	1.9	87.5	100.0
	Asymptomatic	1,187	100.0 (14/14)	78.5–100.0	99.7 (1,169/1,173)	99.1–99.9	1.2	77.8	100.0
	Overall	1,907	100.0 (28/28)	87.9–100.0	99.7 (1,873/1,879)	99.3–99.9	1.5	82.4	100.0
PreservCyt	Symptomatic	1,486	97.9 (44/48)	89.1–99.6	99.9 (1,437/1,438)	99.6–100.0	3.2	97.9	99.9
	Asymptomatic	2,436	95.1 (39/41)	83.9–98.7	100.0 (2,394/2,395)	99.8–100.0	1.7	97.5	99.9
	Overall	3,922	96.6 (86/89)	90.6–98.8	99.9 (3,831/3,833)	99.8–100.0	2.3	97.7	99.9
Endocervical swab	Symptomatic	1,484	100.0 (45/45)	92.1–100.0	99.9 (1,438/1,439)	99.6–100.0	3.0	97.8	100.0
	Asymptomatic	2,464	94.5 (52/55)	85.1–98.1	100.0 (2,408/2,409)	99.8–100.0	2.2	98.1	99.9
	Overall	3,948	97.0 (97/100)	91.5–99.0	99.9 (3,846/3,848)	99.8–100.0	2.5	98.0	99.9
Men, urine	Symptomatic	305	100.0 (82/82)	95.5–100.0	98.7 (220/223)	96.1–99.5	26.9	96.5	100.0
	Asymptomatic	887	100.0 (5/5)	56.6–100.0	99.7 (879/882)	99.0–99.9	0.6	62.5	100.0
	Overall	1,193	100.0 (87/87)	95.8–100.0	99.5 (1,099/1,105)	98.8–99.8	7.3	93.5	100.0
Overall		16,959	97.2 (453/466)	95.3–98.4	99.9 (16,469/16,493)	99.8–99.9	2.7	95.0	99.9

a CC, clinician collected; Cobas, Cobas CT/NG test for use on the Cobas 6800/8800 systems; NPV, negative predictive value; PIS, patient infected status; PPV, positive predictive value; PreservCyt, cervical sample collected in PreservCyt cytology medium; SC, self-collected.

Overall, there were 1,936 CC and 1,906 SC prospective vaginal swab samples. The Cobas sensitivity and specificity performances for the detection of N. gonorrhoeae from CC and SC vaginal swab specimens were the same (100% and 99.7%, respectively) (Table 5).

The rotating PIS analysis results for prospectively collected female urine samples and CC vaginal swabs are shown in Table 6. The sensitivity of Cobas for N. gonorrhoeae detection in female urine samples was higher than the sensitivities of AC2 (*P = *0.012) and CTQ/GCQ (*P = *0.001); all 3 assays showed specificities of ≥99.6% for female urine specimens. The sensitivity of detecting N. gonorrhoeae in prospectively collected CC vaginal swabs was also higher with Cobas than with AC2 and CTQ/GCQ, but the difference was not statistically significant (*P = *1.000). All 3 assays showed specificities of ≥99.5% for vaginal swabs. The sensitivities and specificities for N. gonorrhoeae detection in male urine samples were comparable to those of Cobas, AC2, CTQ/GTQ, and m2000 (≥97.7% and ≥99.2%, respectively; Table 7).

**TABLE 6:** Rotating PIS analysis of sensitivity and specificity of NAATs for detecting Neisseria gonorrhoeae in urine and clinician-collected vaginal swab samples from women^*a*^

Comparison, sample type	No. of samples tested	No. of N. gonorrhoeae PIS-positive samples tested/total no.	Sensitivity (95% CI)	*P* value for comparison with Cobas sensitivity	No. of N. gonorrhoeae PIS-negative samples tested/total no.	Specificity (95% CI)	*P* value for comparison with Cobas specificity
AC2 vs PIS (Cobas and CTQ/GCQ)							
Urine from women	3,853	49/59	83.1 (71.5–90.5)	0.012	3,778/3,794	99.6 (99.3–99.7)	0.002
Vaginal swab (CC)	3,830	56/58	96.6 (88.3–99.0)	1.000	3,752/3,772	99.5 (99.2–99.7)	0.203
CTQ/GCQ vs PIS (Cobas and AC2)							
Urine from women	3,839	46/60	76.7 (64.6–85.6)	0.001	3,778/3,779	100 (99.9–100)	0.625
Vaginal swab (CC)	3,839	56/60	93.3 (84.1–97.4)	0.302	3,777/3,779	99.9 (99.8–100)	0.046

a AC2, Hologic APTIMA Combo 2 CT/NG Assay; CC, clinician collected; Cobas, Cobas CT/NG test for use on the Cobas 6800/8800 systems; CTQ/GCQ, BD ProbeTec CT Q^x^ and GC Q^x^ amplified DNA assays; NAAT, nucleic acid amplification test; PIS, patient infected status.

**TABLE 7:** Rotating PIS analysis of sensitivity and specificity of NAATs for detecting Neisseria gonorrhoeae in urine samples from men^*a*^

Comparison	No. of samples tested	No. of N. gonorrhoeae PIS-positive samples tested/total no.	% sensitivity (95% CI)	No. of N. gonorrhoeae PIS-negative samples tested/ total no.	% specificity (95% CI)
AC2 vs PIS (Cobas, CTQ/GCQ, and m2000)	1,187	86/88	97.7 (92.1–99.4)	1,090/1,099	99.2 (98.5–99.6)
CTQ/GCQ vs PIS (Cobas, AC2, and m2000)	1,187	87/87	100 (95.8–100.0)	1,098/1,100	99.8 (99.3–100.0)
m2000 vs PIS (Cobas, AC2, and CTQ/GCQ)	1,189	87/89	97.8 (92.2–99.4)	1,100/1,100	100 (99.7–100.0)

a AC2, Gen-Probe APTIMA Combo 2 CT/NG assay; Cobas, Cobas CT/NG test for use on the Cobas 6800/8800 systems; CTQ/GCQ, BD ProbeTec CT Q^x^ and GC Q^x^ amplified DNA assays; m2000, Abbot m2000 RealTi*m*e CT/NG assay; NAAT, nucleic acid amplification test; PIS, patient infected status.

## DISCUSSION

This multicenter study evaluated the clinical performance of Cobas at detecting C. trachomatis and N. gonorrhoeae in urogenital samples from men and women. Based on a PIS determined using multiple FDA-approved NAATs, the overall sensitivity and specificity of Cobas for the detection of C. trachomatis were >95% and >99%, respectively, in female urogenital samples and 100% and >99%, respectively, in male urine samples. The overall sensitivity and specificity for detecting N. gonorrhoeae across all female urogenital sample types were >96% and >99%, respectively, and those for male urine samples were 100% and >95%, respectively. Sensitivity and specificity were comparable between symptomatic and asymptomatic individuals for all sample types and were generally consistent across the 9 collection sites. The Cobas assay had a high OPA (>98%) with all predicate FDA-approved NAATs tested for their ability to detect C. trachomatis and N. gonorrhoeae in vaginal swab samples and in urine samples from both women and men.

Previous studies with multiple NAATs have shown that the presence or absence of symptoms does not significantly affect test performance (31, 33, 34). Consistent with these studies, the sensitivities and specificities of Cobas for detecting C. trachomatis and N. gonorrhoeae in our study were comparable between symptomatic and asymptomatic patients. Additionally, Cobas showed a high sensitivity and specificity, regardless of symptomatic status, with a variety of sample types routinely used in clinical practice, including the preferred and minimally invasive vaginal swabs and male urine samples, which are in line with the specimen types recommended by the CDC and European guidelines for C. trachomatis and N. gonorrhoeae detection (11, 12, 22). Of note was the comparable performance of self-collected vaginal swab specimens and clinician-collected vaginal swab specimens. With a performance equivalent to that for clinician-collected specimens, self-collection options decrease barriers to routine STI screening and increase patient satisfaction, thus increasing the likelihood of getting tested and treated in a timely fashion and reducing overall rates of STIs. The ability of Cobas to detect C. trachomatis and N. gonorrhoeae in multiple sample types from both symptomatic and asymptomatic patients indicates that Cobas is suitable for both screening and diagnostic efforts.

We used a rotating PIS, in which 1 of the reference tests served as the investigational device, while the remaining 2 tests (for women) or 3 tests (for men) served as the reference tests in order to construct the PIS. This type of analysis can be helpful in understanding how each of the reference tests used within this study could affect the estimates of assay performance. The rotating PIS also provides a means of showing how each test compares with the others. In this analysis, the sensitivities of Cobas and AC2 for C. trachomatis detection were comparable with both female urine and clinician-collected vaginal swab samples, whereas CTQ/GTQ had a lower sensitivity with both sample types; specificities were comparable across assays. The sensitivity for detecting N. gonorrhoeae in female urine and vaginal swab samples was the highest for Cobas, followed by CTQ/GTQ and AC2; specificities were comparable across the assays. The sensitivities and specificities for detecting both C. trachomatis and N. gonorrhoeae in male urine samples were >97% and >99%, respectively, for all assays.

### Conclusion.

In this multicenter clinical study, Cobas showed a high sensitivity, specificity, PPV, and NPV for the direct, qualitative detection of C. trachomatis and N. gonorrhoeae in a variety of urogenital samples from symptomatic and asymptomatic men and women. The Cobas CT/NG assay for use on the Cobas 6800/8800 systems helps fill the clinical need for a sensitive, high-throughput screening solution to aid in the diagnosis of C. trachomatis and N. gonorrhoeae infections and supports public health efforts to control these infections.

## Supplementary Material

Supplemental file 1
