# Supplementary material for: Evaluation of the Performance of the Cobas CT/NG Test for Use on the Cobas 6800/8800 Systems for Detection of Chlamydia trachomatis and Neisseria gonorrhoeae in Male and Female Urogenital Samples
Source: J Clin Microbiol. 2019 Mar 28;57(4):e01996-18. doi: 10.1128/JCM.01996-18 (PMC6440774; doi:10.1128/JCM.01996-18)
Supplement: Supplemental file 1 [file JCM.01996-18-s0001.pdf]

SUPPLEMENTARY INFORMATION

TABLE S1 Determination of patient infected status from urogenital specimens from women and urine specimens from men

Sample type	BD ProbeTec™ CT/GC Q^x	Hologic APTIMA Combo 2® CT/NG	Abbott RealT*ime* CT/NG	PIS
Women[a]	Urine/vaginal	Urine/vaginal		
	+/+	+/+	NA	Infected
	+/+	+/− or −/+	NA	Infected
	+/− or −/+	+/+	NA	Infected
	+/−	−/+	NA	Infected
	−/+	+/− or −/+	NA	Infected
	−/+	+/−	NA	Infected (urine)<br>Not infected (vaginal)
	+/− or −/+	−/−	NA	Not infected
	+/+	−/−	NA	Not infected
	−/−	+/+	NA	Not infected
	−/−	+/− or −/+	NA	Not infected
	−/−	−/−	NA	Not infected
Men[b]	**Urine**	**Urine**		
	+	+	+	Infected
	+	+	−	Infected
	+	−	+	Infected
	−	+	+	Infected
	−	−	+	Not infected
	−	+	−	Not infected
	+	−	−	Not infected
	−	−	−	Not infected

CT, *Chlamydia trachomatis*; GC, *Neisseria gonorrhoeae*; NG, *Neisseria gonorrhoeae*; NA, not applicable; PIS, patient infected status.

[a] PIS was defined as positive if ≥1 result was positive in each assay; PIS was defined as negative by any other combination of results. PIS was considered indeterminate if results for ≥1 sample type were invalid and the results of the remaining sample types were discordant.

[b] PIS was defined as positive if ≥2 of 3 test results were positive; PIS was defined as negative by any other combination of results. PIS was considered indeterminate if 1 test result was invalid or missing and the other 2 test results were discordant, or if ≥2 of 3 test results were invalid.
